# Anterior Clinoid Meningioma with Optic Chiasm Compression: Seven Years of Preserved Visual Function – A Case Report

**DOI:** 10.1159/cop/acaag014

**Published:** 2026-09-04

**Authors:** Amanda Tran, Jonathan A. Micieli

**Affiliations:** a St. George’s University School of Medicine, St. George, Grenada; b Department of Ophthalmology and Vision Sciences, University of Toronto, Toronto, ON, Canada; c Division of Neurology, Department of Medicine, University of Toronto, Toronto, ON, Canada; d Kensington Vision and Research Centre, Toronto, ON, Canada

**Keywords:** Anterior clinoid meningioma, Skull base meningioma, Optic chiasm compression, Optical coherence tomography

## Abstract

**Introduction:**

Anterior clinoid meningiomas (ACMs) are surgically challenging skull base tumours arising in close proximity to the optic nerve, optic chiasm, and internal carotid artery. In asymptomatic patients, conservative management is well supported, yet the long-term neuro-ophthalmological course of conservatively managed ACMs with documented radiological optic pathway compression remains poorly characterized.

**Case Presentation:**

A 77-year-old woman was referred to neuro-ophthalmology following an incidental discovery of a right anterior clinoid mass. Magnetic resonance imaging (MRI) confirmed uniformly enhancing extra-axial mass compressing the optic chiasm and displacing the right optic nerve medially. She was asymptomatic with normal visual acuity, Humphrey visual field, and optical coherence tomography (OCT) at baseline. Conservative management was elected given her age, significant comorbidities, and absence of symptoms. Over 7 years of follow-up visits every 6 months, she remained visually asymptomatic with stable visual acuity, visual fields, and serial OCTs of the retinal nerve fibre layer and ganglion cell complex, both remaining above published thresholds associated with unfavourable visual outcomes in ACMs. Serial MRIs confirmed tumour stability throughout.

**Conclusion:**

This case documents one of the longest reported periods of structured neuro-ophthalmological surveillance in a conservatively managed ACM with radiological chiasmal compression, demonstrating sustained preservation of visual function and structural optic nerve integrity over 7 years. Serial OCT should be integrated into the longitudinal surveillance of patients with ACMs when intervention is deferred.

## Introduction

Anterior clinoid meningiomas (ACMs) are a distinct subgroup of skull base meningiomas that arise from the anterior clinoid process in close proximity to the optic nerve, optic chiasm, and internal carotid artery, making them among the most surgically challenging intracranial tumours to safely remove [1]. Al-Mefty’s [1] classification stratifies ACMs according to their site of origin relative to the carotid cistern: group I tumours arise proximal to the cistern, lack an intervening arachnoid membrane between the tumour and the carotid artery, and carry the greatest surgical risk, while group II tumours arise from the superior or lateral aspect of the clinoid above the cistern, preserving an arachnoid plane that makes dissection feasible. Gross total resection is achievable in only approximately 12% of the most difficult subgroup, compared with over 90% of group II lesions, and incomplete resection carries a substantially higher risk of recurrence [2].

In asymptomatic patients, conservative management is well supported. Nakamura et al. [3] found a mean tumour volume doubling time of 21.6 years in conservatively managed meningiomas, with calcification and T2 hypointensity or isointensity signals predicting slower growth. The radiological features of our patient’s tumour, projecting superiorly from the clinoid without cavernous sinus involvement or vascular encasement, are consistent with group II, though intraoperative confirmation was never sought given her clinical stability.

Despite the frequency with which ACMs are managed conservatively, the long-term neuro-ophthalmological course of patients with documented radiological optic pathway compression who remain asymptomatic is poorly characterized. Most published series focus on surgical or radiosurgical outcomes, leaving a gap in the literature regarding what structured serial monitoring looks like in practice and what it can demonstrate over time. We present a case of a conservatively managed ACM with radiological chiasm compression and 7 years of serial neuro-ophthalmological surveillance data.

## Case Report

A 77-year-old woman was referred to neuro-ophthalmology in August 2019 after a right anterior clinoid mass was incidentally found on computed tomography imaging of her brain following a mechanical fall. Her medical history included atrial fibrillation, dyslipidaemia, and osteoporosis, and her medications included apixaban, atorvastatin, ezetimibe, and risedronate. She had no visual symptoms, reporting no blurred vision, diplopia, ocular pain, or perceived visual field loss, and had undergone an uneventful cataract surgery 6 months earlier.

Magnetic resonance imaging (MRI) of the brain in January 2019 demonstrated a well-defined, 17 mm, uniformly enhancing extra-axial mass with a dural base centred on the superior surface of the right anterior clinoid process, with imaging feature typical of a meningioma. The mass displaced the optic chiasm and cisternal segment of the right optic nerve medially, extending to but not into the right optic canal. The orbits were clear.

At baseline, best-corrected visual acuity was 20/30 in the right eye (OD) and 20/20 in the left eye (OS). Confrontation fields were full bilaterally. Pupils were equal and reactive with no relative afferent pupillary defect. Optic nerve heads and maculae appeared normal, and extraocular motility and alignment were intact. Humphrey visual field (HVF) was normal, and optical coherence tomography (OCT) of the retinal nerve fibre layer (RNFL) and ganglion cell complex (GCC) showed values within normal limits bilaterally (Fig. 1). Given her age, comorbidities, and absence of symptoms, neurosurgery elected to manage the tumour conservatively.

**Fig. 1.**
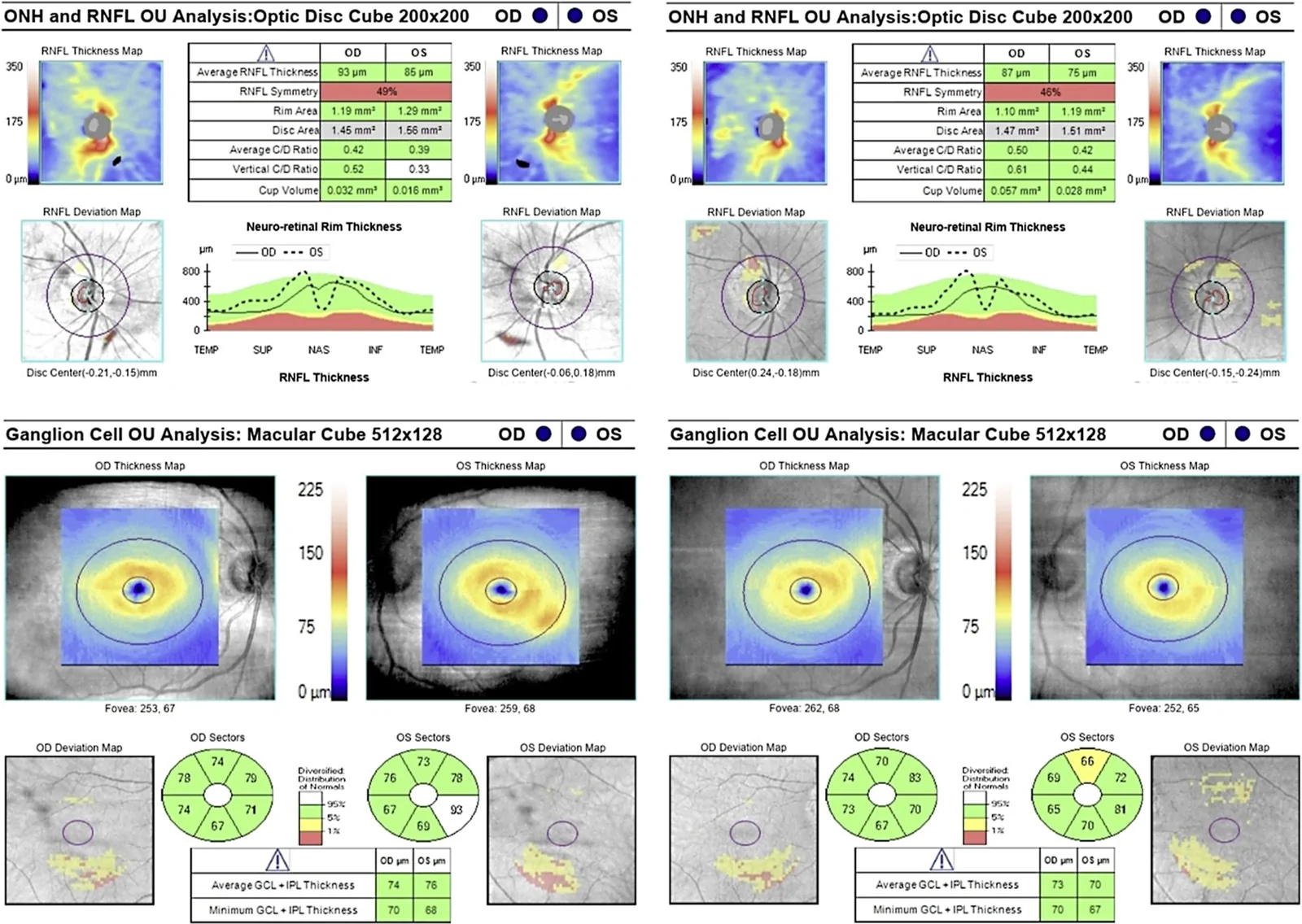
OCT RNFL (top) and GCC (bottom) at baseline (2019, left) and most recent follow-up (2026, right). Average RNFL thickness in the right eye measured 93 µm and 87 µm, respectively, and average GCC thickness in the right eye measured 74 µm and 73 µm, respectively.

The patient attended surveillance visits every 6 months over the following 7 years. At each assessment, she remained without new visual complaints, and best-corrected visual acuity was 20/20 bilaterally at all subsequent visits. HVF was stable throughout, and serial OCTs showed no interval thinning of the RNFL or GCC (Table 1). Optic nerve heads remained pink and sharp without pallor or oedema, and no relative afferent pupillary defect was detected at any visit. Average RNFL thickness in OD measured 93 µm at baseline and 87 µm at the most recent follow-up, and average GCC thickness measured 74 µm and 73 µm, respectively (Fig. 1). Intermittent epiphora and blepharitis were managed conservatively. At the most recent assessment in April 2026, examination was stable.

**Table 1.** Serial neuro-ophthalmological surveillance findings over 7 years

​	HVF		OCT RNFL		OCT GCC	
	mean deviation		µm		µm	
	OD	OS	OD	OS	OD	OS
August 2019	−1.12	−1.46	93	85	74	76
September 2020	−2.07	−2.06	91	89	74	75
March 2021	0.72	−0.10	86	76	74	74
October 2021	−0.64	−1.75	–	–	–	–
April 2022	−0.70	−2.21	84	79	75	67
January 2023	−1.78	−2.26	81	76	74	73
July 2023	−0.52	−2.53	79	66	71	72
January 2024	−1.22	−3.90	81	82	72	72
February 2025	1.25	−2.27	83	78	74	73
August 2025	1.92	−2.19	81	78	72	70
April 2026	−0.58	−5.15	87	75	73	70

HVF mean deviation is reported in decibels. RNFL and GCC values represent average thickness in micrometres. OCT was not performed in October 2021. OD, right eye; OS, left eye; HVF, Humphrey visual field; OCT, optical coherence tomography; RNFL, retinal nerve fibre layer; GCC, ganglion cell complex.

Serial MRIs confirmed tumour stability throughout the surveillance period. The October 2022 study showed the lesion measuring approximately 1.9 cm, projecting superiorly from the clinoid and abutting the supraclinoid internal carotid artery and right anterior cerebral artery without encasement. The optic chiasm and cisternal right optic nerve remained displaced medially along the mass. The July 2024 MRI confirmed an unchanged size and configuration measuring 1.8 × 1.7 × 1.8 cm, and no change in the degree of mass effect on the right optic nerve and chiasm. The July 2025 MRI again demonstrated stability at approximately 1.8 cm, with mild hyperostosis of the underlying bone and abutment of the right carotid termination and A1/M1 segments without carotid narrowing or chiasmal oedema (Fig. 2). In July 2024, the patient received a new diagnosis of lung cancer treated by a wedge resection, and serial neuroimaging remained consistent with stable meningioma without features suggesting metastatic transformation.

**Fig. 2.**
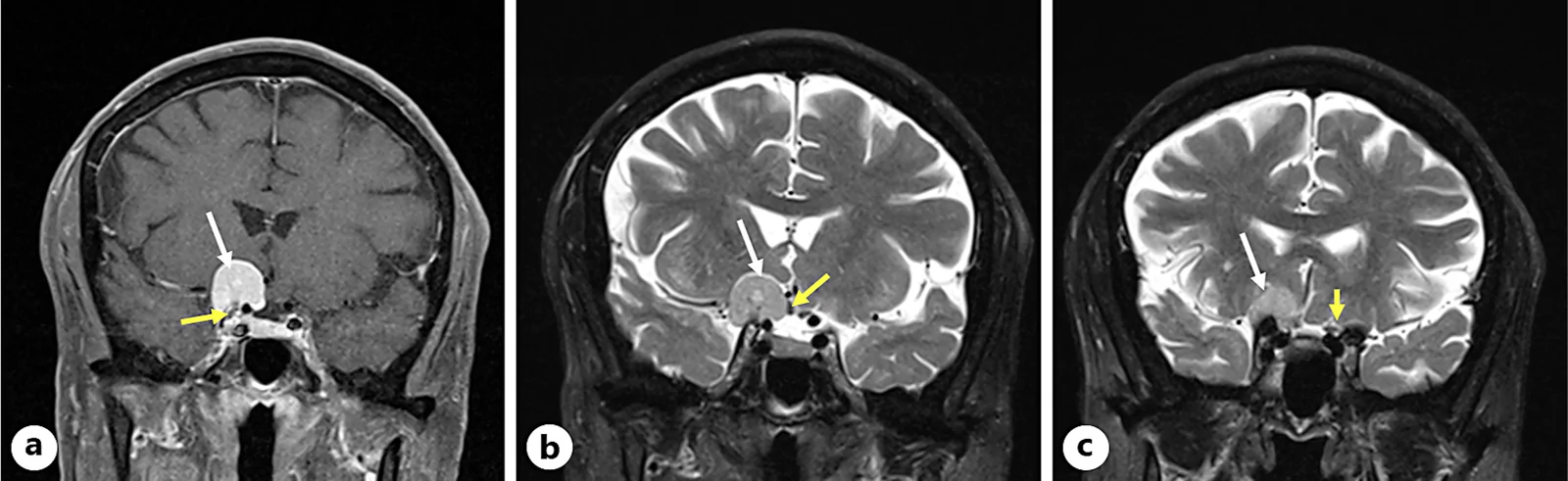
Coronal MRI of the brain demonstrating a right ACM (white arrows) compressing the ipsilateral optic nerve (yellow arrows). **a** Post-contrast T1-weighted sequence demonstrating the uniformly enhancing extra-axial mass. **b**, **c** T2-weighted sequences. All images are from the most recent MRI study (July 2025).

This case report follows the CARE guidelines. The CARE Checklist is attached as online supplementary material (for all online suppl. material, see https://doi.org/10.1159/cop/acaag014).

## Discussion

The key feature of this case is that despite clear MRI evidence of the tumour compressing the optic nerve and chiasm, our patient never developed any visual symptoms or signs over 7 years of follow-up. Cohen-Cohen et al. [4] identified RNFL ≤80 µm and GCC ≤70 µm as predictors of unfavourable visual outcomes in ACMs, though these thresholds were derived in a surgical cohort to guide the timing of intervention. Our case applies these values in a different setting, using them as benchmarks during long-term surveillance rather than as preoperative predictors. By remaining above both thresholds throughout follow-up, our patient’s OCTs provided objective evidence that progressive axonal loss was not occurring and that the optic nerve was tolerating the compression without significant structural damage, a finding that clinical examination and visual fields alone cannot reliably detect.

The mechanisms underlying preserved visual function despite sustained compression are not fully understood; however, several factors may have contributed. Visual loss in chiasmal compression is thought to result from a combination of conduction blocks, demyelination, ischemia, and retrograde and anterograde degeneration [5]. The slow growth of this tumour, evidenced by radiological stability across 7 years, may have avoided the acute ischemic and mechanical insults that precipitate these processes, allowing the optic chiasm and right optic nerve to accommodate the mass gradually. Experimental work suggests that the optic projection is not passive under chronic stress; rather, it employs adaptive responses, including enhanced retinal ganglion cell excitability and redistribution of the metabolic resources by the astrocytes to preserve axonal function [6]. The pattern of compression in our case may also be relevant. The tumour displaced rather than encased the optic nerve, and radiographic encasement has been identified as a predictor of unfavourable visual outcomes in ACMs [4]. Experimental and histological studies of chronic compressive and glaucomatous optic neuropathy have shown that larger nerve fibres tend to preferentially degenerate [7], with relative preservation of the smaller fibres, which may explain how central visual acuity and field sensitivity can be maintained despite structural compression. Notably, this dissociation is not unique to our patient. In a series of 81 conservatively managed pituitary adenomas with optic nerve compression, only 17% developed visual deterioration over a median of 5 years [8], suggesting that the optic apparatus can often tolerate slow compression without functional visual loss. These mechanisms remain speculative in a single case; however, together they offer a plausible explanation for the structure-function dissociation observed.

While the natural history studies supporting conservative management focus primarily on tumour growth [3], and the broader literature reports predominantly surgical or radiosurgical outcomes [2], comparatively little attention has been paid to the structural integrity of the optic pathway over time in patients managed conservatively. Quantitative OCT data across an extended surveillance period are particularly scarce. This case helps address that gap by documenting 7 years of serial RNFL and GCC measurements alongside stable visual fields and imaging, offering a concrete picture of what preserved optic nerve function looks like structurally when an ACM compressing the optic nerve is conservatively managed rather than resected.

This case illustrates three principles. Firstly, radiological compression of the optic nerve and chiasm does not always cause visual dysfunction, and this discordance can persist for many years. Secondly, regular neuro-ophthalmological follow-up combining serial HVF and OCT of the RNFL and GCC provides a sensitive and objective means of detecting subclinical change before irreversible visual loss occurs. Thirdly, in elderly patients with significant comorbidities, conservative management is not a suboptimal approach; rather, it is a well-supported strategy that weighs the risks of surgical intervention against the typically indolent natural history of incidental meningiomas. In our patient, the concurrent diagnosis of lung cancer further added diagnostic complexity, reinforcing the importance of continued imaging surveillance to ensure the intracranial lesion remained consistent with a stable meningioma.

This report has a few limitations. As a single case, its findings cannot be generalized. The diagnosis of meningioma was based on characteristic imaging rather than histopathology, as no biopsy was performed. Additionally, the Al-Mefty [1] classification was inferred radiologically without intraoperative confirmation.

To our knowledge, this represents one of the longest documented periods of structured neuro-ophthalmological surveillance in a conservatively managed ACM with radiological chiasmal compression. Our case extends the evidence base for conservative management of incidentally discovered ACMs compressing the optic nerve and chiasm and highlights the clinical utility of integrating serial OCT into the longitudinal surveillance of patients with ACMs in whom intervention is deferred.

## Statement of Ethics

This retrospective review of patient data did not require ethical approval in accordance with local/national guidelines. The patient provided written informed consent for the publication of this case report and the accompanying images.

## Conflict of Interest Statement

The authors have no conflicts of interest to declare.

## Funding Sources

This study was not supported by any sponsor or funder.

## Author Contributions

A.T.: literature review, data collection, and writing original draft. J.A.M.: supervision, conceptualization, reviewing, and editing.

## Data Availability

The data supporting this study’s findings are not publicly available due to privacy and security reasons but can be obtained from the corresponding author upon reasonable request.
